# Retinal fingerprints of ALS in patients: Ganglion cell apoptosis and TDP-43/p62 misplacement

**DOI:** 10.3389/fnagi.2023.1110520

**Published:** 2023-03-16

**Authors:** Natalia Pediconi, Ylenia Gigante, Silvia Cama, Martina Pitea, Lorenza Mautone, Giancarlo Ruocco, Silvia Ghirga, Silvia Di Angelantonio

**Affiliations:** ^1^Center for Life Nano- and Neuro-Science of Istituto Italiano di Tecnologia (IIT), Rome, Italy; ^2^D-Tails s.r.l., Rome, Italy; ^3^Department of Physiology and Pharmacology, Sapienza University of Rome, Rome, Italy; ^4^Department of Physics, Sapienza University of Rome, Rome, Italy

**Keywords:** retina, ALS, neurodegeneration, biomarkers, protein inclusions, neurons, microglia, TDP-43

## Abstract

**Introduction:**

Amyotrophic lateral sclerosis (ALS) is a fatal neurodegenerative disease characterized by the progressive loss of motor neuron function. Although ophthalmic deficits are not considered a classic symptom of ALS, recent studies suggest that changes in retinal cells, similar to those in the spinal cord motor neurons, have been observed in postmortem human tissues and animal models.

**Methods:**

In this study, we examined by immunofluorescence analysis the retinal cell layers of sporadic ALS patients in post-mortem retinal slices. We evaluated the presence of cytoplasmic TDP-43 and SQSTM1/p62 aggregates, activation of the apoptotic pathway, and microglia and astrocytes reactivity.

**Results:**

We found in the retinal ganglion cell layer of ALS patients the increase of mislocalized TDP-43, SQSTM1/p62 aggregates, activation of cleaved caspase-3, and microglia density, suggesting that retinal changes can be used as an additional diagnostic tool for ALS.

**Discussion:**

The retina is considered part of the central nervous system, and neurodegenerative changes in the brain may be accompanied by structural and possibly functional changes in the neuroretina and ocular vasculature. Therefore, using *in vivo* retinal biomarkers as an additional diagnostic tool for ALS may provide an opportunity to longitudinally monitor individuals and therapies over time in a noninvasive and cost-effective manner.

## 1. Introduction

The eye has always been considered a window to the soul. Today this saying is more relevant than ever, given the growing evidence of retinal manifestations in a range of diseases typical of the central nervous system (CNS). Indeed, being an integral part of the CNS, the retina shares many mechanisms with the brain and often mirrors CNS disease processes. Indeed, the brain, spinal cord, and retina share a common embryological origin and appear to be affected by the same neurodegenerative processes (Kavcic et al., 2011; London et al., 2013; Chang et al., 2014; Lee et al., 2014; Javaid et al., 2016; Mancino et al., 2019).

Visual abnormalities and signs of retinal pathology have been reported in patients with Alzheimer’s disease (AD), Parkinson’s disease (PD), and Huntington’s disease, multiple sclerosis and amyotrophic lateral sclerosis (ALS; Gupta et al., 2021). In addition, the eye offers a unique opportunity for direct observation of nerve tissue due to its transparent optical media. For these reasons, the retina is now called the window to the brain (Gupta et al., 2021). This has prompted interest in using retinal imaging to diagnose and monitor neurodegenerative diseases *in vivo* with noninvasive techniques, bringing with it the need to identify biomarkers to diagnose and monitor neurological conditions. However, to rationally implement retinal imaging biomarkers in clinical practice, it is necessary to understand the cellular and molecular processes occurring in the retina and their relationship to brain pathology. In recent years, progress has been made in understanding neuropathological changes in the retina of AD and PD patients. Alzheimer’s disease patients and animal models show retinal alterations, such as thinning of the ganglion cell layer, the presence of protein aggregates, glial activation, and alterations in the vasculature (den Haan et al., 2017; Koronyo et al., 2017; Grimaldi et al., 2018, 2019); in Parkinson’s disease, in addition to motor symptoms, visual function is also impaired, and postmortem evidence reports reduced retinal dopamine content and accumulation of α-synuclein (Altintaş et al., 2008).

In this context, even for ALS, traditionally considered a disease of the pure motor system, there is a growing body of evidence supporting the involvement of other non-motor systems, including the eyes (Nepal et al., 2022). Indeed, the traditional view that the visual system is spared in ALS (Ringelstein et al., 2014) has recently been challenged: atrophy of retinal layers (Moss et al., 2012; Fawzi et al., 2014; Rohani et al., 2018) has been reported, and retinal thinning may reflect an underlying neurodegenerative process (Volpe et al., 2015).

Amyotrophic lateral sclerosis, the most common form of motor neuron disease, is a progressive and ultimately fatal condition, with an average life expectancy of 2–3 years after diagnosis (Hübers et al., 2016).

Despite increasing knowledge, so far the diagnosis of ALS is still made by exclusion and often delayed, as there is no specific test that can confirm the disease (Marin et al., 2018). Several biomarkers have recently been identified, but none has already been implemented in clinical practice (Bakkar et al., 2015). Thus, there is an urgent unmet need to find effective tools to aid in the accurate diagnosis, stratification, and monitoring of disease progression in ALS patients.

While only a small percentage of ALS cases have a familial origin (fALS), attributable to selective mutations in causative and heritable genetic (SOD1, TARDBP, C9orf72, etc.), the vast majority of cases are believed to have a sporadic origin (sALS), in which genes and environmental factors interact. However, a number of cellular pathways/functions have been associated with the pathogenesis of sALS and fALS, such as RNA processing, dysfunctional protein homeostasis, endoplasmic reticulum stress, dysfunctional mitochondria and/or energy metabolism, increased oxidative stress, altered axonal transport, and dysfunction of RNA-binding proteins (Zhao et al., 2022).

In particular, the presence of protein aggregate inclusions in spinal cord tissue is a hallmark of ALS, present in 97% of familial and sporadic cases. These inclusions may involve different components such as neurofilaments, ubiquitin, RNA-binding proteins such as TDP-43 (coded by TARDBP), and the antioxidant enzyme superoxide dismutase 1 (SOD1). In most cases, these inclusions are positive for ubiquitin/p62, ubiquilin-2, and p-TDP-43 (Arai et al., 2006; Neumann et al., 2006). In familial and sporadic cases, TDP-43 observed in autopsy tissue is found in cytoplasmic aggregates and is aberrantly phosphorylated, ubiquitinated, and cleaved. TDP-43 is normally a primarily nuclear protein, with roles in mRNA translation, splicing and retention of cryptic exons, nuclear export, and axon growth. Cytoplasmic mislocation of TDP-43 is considered to be an important pathogenic mechanism in ALS, likely affecting several biological processes that reduce neuronal viability. TDP-43-positive protein aggregates in ALS often also contain the p62 protein. The consistent presence of p62 in pathological inclusions and the identification of Sequestosome 1 (SQSTM1)/p62 mutations as a rare cause of ALS suggest a role in pathogenesis (Falchetti et al., 2009 and reviewed in Rea et al., 2013).

Retinal involvement in ALS has only been demonstrated in familial forms using *SOD1* and *C9orf72* mice and in a patient with ALS and expansion of the GGGGCC hexanucleotide repeat in the C9orf72 gene (C9-ALS). Notably, *SOD1* mutant mice show retinal cell degeneration and protein aggregates have been reported to parallel spinal cord degeneration (Hashizume et al., 2008; Ringer et al., 2017; Soldatov et al., 2021). In addition, *C9orf72* mutant mice show retinal inclusions of p62 in the inner layer (Soldatov et al., 2021). In contrast, the mouse model of ALS FUS revealed no significant structural changes in the retina (Soldatov et al., 2021). Regarding human tissues, histopathological examination of the retina, optic nerve, and CNS of a C9orf72 ALS patient demonstrated p62-positive and pTDP-43-negative perinuclear inclusions in the inner nuclear layer of the retina and CNS. These cytoplasmic inclusions of ALS-related misfolded proteins suggest that the inner layer is the site of ALS-associated neurotoxicity (Rojas et al., 2020). However, data on sporadic ALS and other human tissues are not available.

To better understand and identify new molecular targets for a more comprehensive panel of putative biomarkers for ALS diagnosis, we performed immunostaining on human retinas obtained from sporadic ALS patients and age-matched controls. We report that the retinas of ALS patients show, in the granule cell layer, p62 inclusions, cell mislocalisation of TDP-43, and neuronal apoptosis, together with the increase in microglia density. These results highlight that retinal protein aggregates and cellular markers are targets to be considered for ALS diagnosis.

## 2. Materials and methods

### 2.1. Human samples

Human retinal slices from sporadic ALS patients and age matched controls were purchased from Human Eye Biobank for Research, St Michael Hospital, Toronto, Canada. Prior to death, donors signed informed consent for autopsy, use of tissue and medical records for research purposes. No documented history of eye disease was reported for the ALS or control cases. The use of Human tissue has been approved by the Ethics Committee of Fondazione Santa Lucia I.R.C.C.S. to GR.

### 2.2. Immunofluorescence

Retinal slices were deparaffinized using a standard protocol. Briefly, slices were washed in Xylene (three washes, 5 min each) and after rehydration in decreasing concentrations of ethanol (100, 95, 70 and 50%, two washes, 10 min each), then rinsed twice in distilled water and washed with phosphate buffered saline (PBS) solution (three washes, 5 min each). Antigen retrieval has been performed by incubating slices at 90°C for 45 min in a solution containing sodium citrate pH 6 (Sigma Aldrich). Slices were allowed to cool down at room temperature for 20–30 min, washed twice in PBS and then incubated for 1 h in a blocking solution containing 5% goat serum and 0.3% Triton in PBS. Primary antibodies diluted in blocking solution were incubated overnight at +4°C: anti-TDP-43 (Abcam, #ab109535, clone EPR5810, 1:250); anti-SQSTM1 (Santa Cruz biotech. #sc-28359, clone D3, 1:200); anti-Cleaved Caspase-3 (Cell signaling # 9661 s, clone Asp175, 1:100); anti-β-tubulin-III (Sigma #T2200, clone Aa441-450, 1:1,000); anti-Iba1, (FUJIFILM WAKO # 019–19741, 1:400); anti-GFAP (Millipore #MAB360, clone GA5; 1:400); and anti-pTDP-43 ser409 (COSMOBIO, 1:500). Negative controls were performed by omitting the primary antibody. On the following day, samples were washed in PBS (3 × 5 min) and then incubated for 45 min with Hoechst (1:1,000) and secondary fluorescent antibody. After 3 × 10 min washes in PBS slices were incubated with Sudan Black (0.3% in EtOH 70%) for 10 min to reduce the level of autofluorescence. The samples were dipped in EtOH 70%, distilled water and mounted using an antifade Fluorescence Mounting Medium (Dako-Agilent). Images were acquired with either a Confocal Laser Scanning or a Confocal Spinning Disk microscope.

### 2.3. Laser scanning acquisitions

For conventional Confocal Laser Scanning analysis of retinal slices, images (size 1,024 × 1,024 pixel) were acquired with a Laser Scanning Confocal microscope (FV10i fluoview, Olympus) equipped with a 60X objective water immersion objective (NA 1.2). Three wavelength diode lasers have been used (405, 473, and 559 nm). When possible, to minimize any putative bias in sampling, six images were collected from the retina of each patient (usually three from the upper and three from the lower retina). For each image 20–25 stacks (0.5 μm step each) were acquired and analysis was performed with ImageJ software (2.1.0/1.53c).

### 2.4. Confocal spinning disk acquisition

Acquisition of the images was also performed using a Nikon Eclipse Ti equipped with a X-Light V3 spinning disk (CrestOptics). The images were acquired with Metamorph software version 7.10.2. (Molecular Devices) using a 60x PlanApo l oil objective (1.42 numerical aperture) and sectioning the slice in Z with a step size of 0.3 μm each. The acquisitions obtained were analyzed using the ImageJ software.

### 2.5. Analysis of TDP-43

Fluorescence images of TDP-43 and Hoechst staining were analyzed through a custom algorithm implemented in MATLAB environment. Images were pre-processed as follows: a moving average filter (3 × 3 pixels window) reduced the noise, and a histogram shape-based method automatically identified the background level beyond the peak of the smoothed histograms. Furthermore, for Hoechst signals, a morphological opening operation (disk ray = 6 pixels) was performed to remove small objects and thin lines from the images while preserving nuclei shape. Corrected images were binarized by combining a clustering method with a locally adaptive thresholding. The pixels of the image were divided into two groups (signal and background) by computing a two-means clustering of the image. Additionally, adaptive thresholds, based on the local mean intensity in the neighborhood of each pixel, are necessary for different lighting conditions in different areas. The union of the two masks returns the binary image of Hoechst and TDP-43 signals used to evaluate the spatial distribution of TDP-43 with respect to the cell nucleus. First, the total area covered by Hoechst signal was divided by the area of a single nucleus to estimate the number of cells in the field of view; then the areas covered by TDP-43 signal outside and inside nuclei, normalized by the number of cells, were computed and compared in control and ALS conditions.

### 2.6. Co-localization of pTDP-43 signals with TDP-43

Co-localization between cytoplasmatic TDP-43 with its phosphorylated form pTDP-43 was evaluated by examining fluorescence co-stained images acquired by Confocal Laser Scanning. Images analysis was performed in a Matlab environment. Firstly, we exploit the Hoechst signal to select the region of interest outside nuclei, as explained in the 2.5 paragraph. We then analyzed the average intensity projection of TDP-43 and pTDP-43 stack images within the selected area. We subtracted the local background and applied H-minima transform to suppress non-specific signals, then we binarized images using Otsu’s method, which chooses a threshold that minimizes the intraclass variance of the thresholded black and white pixels. Finally, we calculated Manders overlap coefficient to measures the fraction of the cytoplasmatic TDP signal covered by the pTDP signal.

### 2.7. Co-localization of SQSTM1/p62 signals with TDP-43

To estimate co-localization between p62 and TDP-43 staining, we calculated Manders overlap coefficient, which measures the fraction of the p62 signal coincident with the TDP-43 signal. Quantification was performed after subtracting local background to avoid high co-localization values due to the non-specific signal (i.e., photoreceptor autofluorescence).

### 2.8. Analysis of SQSTM1/p62 aggregates

Images were acquired by Confocal Spinning Disk microscopy, a Z-projection based on the maximal intensity signal was obtained and after threshold setting, fluorescence intensity value has been recorded. Data are expressed as mean fluorescence integrated density/region of interest (ROI) limited to threshold.

### 2.9. Analysis of neurodegeneration

Images of slices immunostained for cleaved Caspase-3, an effector enzyme of the apoptotic pathway, were acquired by Confocal Spinning Disk microscopy. Number of positive cells were manually counted within each image. Results are expressed as the number of cleaved Caspase-3 positive cells divided by the total number of ganglion neurons counted in the region of interest (ROI) and then calculated as percentage of degenerating ganglion cells in each slice.

### 2.10. Microglia density and astrogliosis analysis

To quantify the amount of GFAP and Iba1 signals (images acquired by Confocal Spinning Disk), we used the following methods designed in a Matlab environment. Briefly, for GFAP analysis, we drew a polygonal region of interest (ROI) around the inner nuclear layer on the average intensity projection of the Hoechst channel. Then, the average intensity projection for the GFAP channel stacks was analyzed: after local background subtraction and H-minima transform application, images were binarized using Otsu’s method. The area of the binarized image within the first ROI was measured, normalized on the first ROI, and thus expressed as percentage of area covered. For Iba1 immunostaining we manually count the number of iba1 positive cells over the field of view.

### 2.11. Statistics and data analysis

In order to avoid bias due to thresholding, data have been analyzed by two different experimenters by using a fixed threshold in one case (Triangle method in ImageJ), or using for each image the best threshold according to the experimenter’s judgment. Both methods gave similar results thus reducing the possibility of any bias introduction. Data are shown as the Mean +/− SEM. Statistical significance between controls and ALS patients was assessed with the nonparametric Mann–Whitney U test or T-test as indicated. A *p* value <0.05 was considered significant. All statistical analyses were performed using Prism9 software.

## 3. Results

### 3.1. Presence of TDP-43 and p62 protein aggregates in the retina of ALS patients

The accumulation of TDP-43 and p62 aggregates in retinal tissue has been reported in genetic mouse models of ALS (Rojas et al., 2020) suggesting a putative use of the eye as a valuable structure for the study and diagnosis of ALS. Using human retinal slices from ALS patients and age-matched controls (samples were obtained from the Human Eye Biobank at St. Michael’s Hospital), we analyzed the presence of protein inclusions in the retinal layers.

We analyzed retinal cross sections from 10 clinically and neuropathologically confirmed ALS patients (mean age 65 ± 2 years; range 54–74 years; five females and five males) and 11 healthy controls (mean age 62 ± 2 years; range: 48–70 years; six females and five males). To analyze the presence of cytoplasmic TDP-43 inclusions, we stained retinal slices with an antibody against total TDP-43. We quantified the amount of TDP-43 inclusions measuring the areas covered by TDP-43 signal inside and outside the nucleus (stained with Hoechst) and normalized by the number of cells.

**Figure 1:**
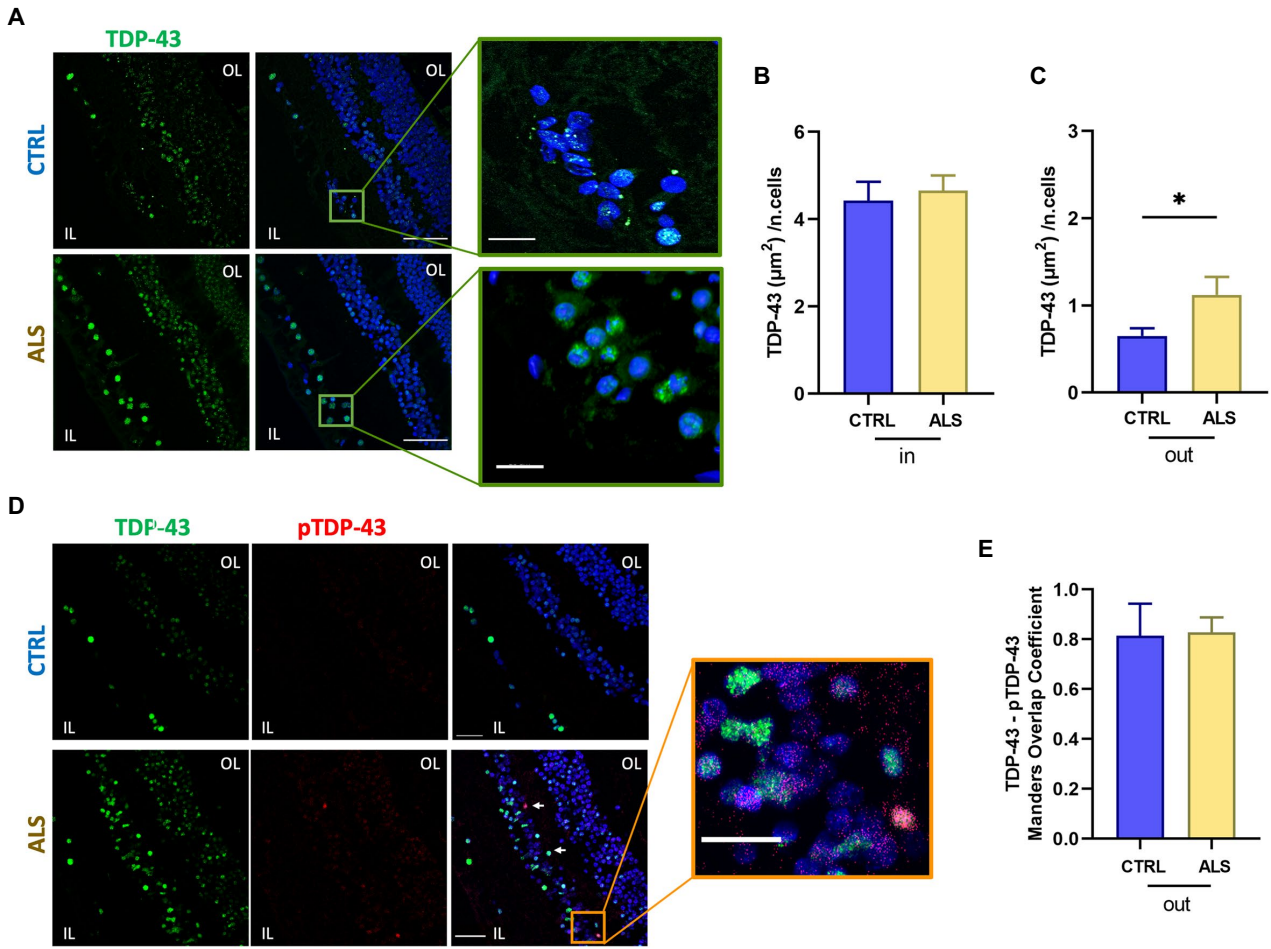
TDP-43 inclusions are mislocalized in the human amyotrophic lateral sclerosis (ALS) retina. **(A)** Representative images of retinal slices from ALS patients and healthy controls immunolabeled with anti-TDP-43 (green) and Hoechst for nuclei visualization (blue); scalebar 50 μm. Zoom: Imaris 3D reconstruction of TDP-43 staining in the ALS retina at higher magnification showing TDP-43 staining outside the nucleus; bar 10 μm. **(B)** Bar chart showing the quantification of the area covered by TDP-43 signal inside the nucleus (in) normalized by the number of cells (ALS *n* = 57/7 fields/individuals; CTRL *n* = 50/6 fields/individuals). **(C)** Bar chart showing the quantification of the area covered by TDP-43 signal outside the nucleus (out) normalized by the number of cells (^*^p < 0.05 ALS vs. CTRL, Mann–Whitney U test; ALS *n* = 57/7 fields/individuals; CTRL *n* = 50/6 fields/individuals). **(D)** Representative immunostaining of anti-TDP-43 (green), anti-pTDP-43 (red), and Hoechst for nuclei visualization (blue) in ALS and control retinal slices; scale bar 50 μm. Arrows indicate co-localized signals. Zoom: Co-localization of TDP-43 and pTDP-43 in the ALS retinal slice; scalebar 20 μm. IL, inner layer; OL, outer layer. **(E)** Bar chart displaying the Manders Overlap coefficient of pTDP-43 and TDP-43 signals in the cytoplasmic region. Note that the amount of cytoplasmic TDP-43 is significantly higher in ALS patients **(C)**.

Nuclear staining of TDP-43 gave a strong and diffuse staining in all retinal layers in both ALS patients and control subjects (Figures 1A,B; ALS: 4.6 ± 0.3, *n* = 57/7 fields/individuals; controls 4.4 ± 0.4, *n* = 50/6 fields/individuals; *p* = 0.9). TDP-43 immunoreactivity was strikingly and significantly higher in the cytoplasmic compartment of retinal cells of ALS patients compared to age matched controls (ALS: 1.2 ± 0.2, *n* = 57/7 fields/individuals; controls 0.65 ± 0.09, *n* = 50/6 fields/individuals *p* < 0.05; FOV = 400 μm^2^), thus confirming the accumulation of TDP-43 mislocalization in ALS patients retina (Figure 1C).

We analyzed the colocalization of TDP-43 with its phosphorylated form pTDP-43 at Ser409 (Figure 1D), because in ALS motor neurons, insoluble pathological inclusions of TDP-43 consist mainly of cytoplasmic deposition of C-terminally phosphorylated TDP-43 (Neumann et al., 2009). Quantification of co-localized signals showed that pTDP-43 co-localized with the cytoplasmic TDP-43 staining (Figure 1E), in both control and ALS patients’ retina giving similar Manders coefficients (ALS: 0.82 ± 0.02, *n* = 6 patients; controls: 0.81 ± 0.05, *n* = 6 individuals). It has to be noticed, however, that both cytoplasmic TDP-43 and pTDP-43 signals were significantly higher in ALS retina compared to age matched controls (see Figures 1C,D bottom).

**Figure 2:**
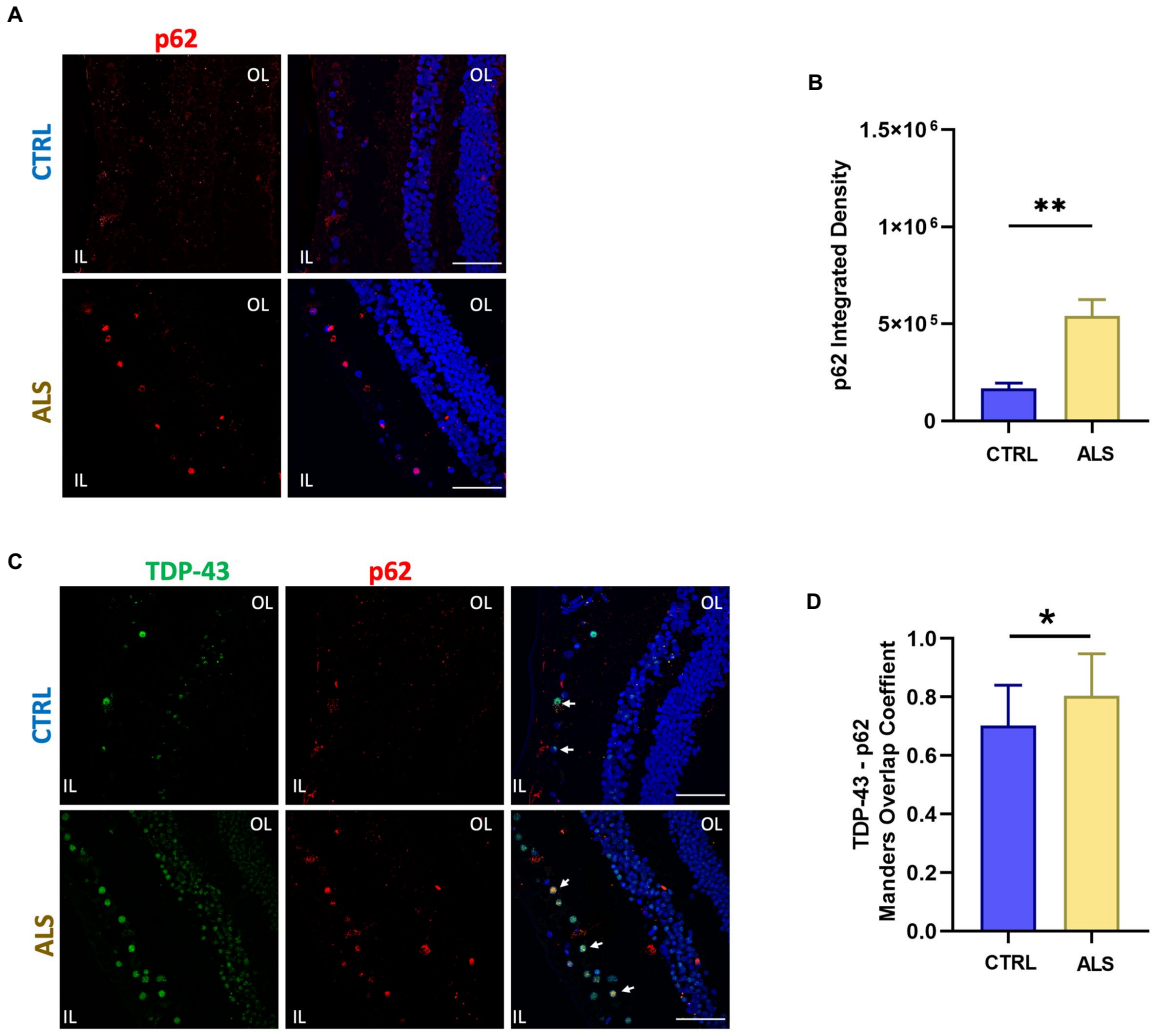
SQSTM1/p62 aggregates accumulate in the human ALS retina. **(A)** Representative immunofluorescence images of anti-p62 staining (red) on human retinal slices from ALS patients and healthy controls. Hoechst was used to stain nuclei (blue); scale bar 50 μm. **(B)** Bar chart showing the quantification of p62 aggregates accomplished by integrated density/field of view (^**^p < 0.01 ALS vs. CTRL; Mann–Whitney U test; ALS *n* = 59/7 fields/individuals; CTRL *n* = 65/6 fields/individuals). **(C)** Representative immunostaining of TDP-43 (green) and p62 (red) in human retinal slices. Arrows indicate co-localized signals. **(D)** Bar graphs representing the co-localization of p62 and TDP-43 positive inclusions in ALS retinal slices calculated using Manders overlap coefficient (ALS: *n* = 17/4; CLTR: 14/4 fields/individuals; ^*^p < 0.05).

In all ALS patients analyzed we detected retinal p62 immunoreactivity and deposits both in the inner and outer layers (Figure 2A). Sparse and diffuse retinal p62 deposits were found occasionally also in control slices (Figure 2A top-left). Staining of p62, evaluated as p62 signal integrated density in a region of 400 μm^2^, was significantly higher in ALS patients (ALS: 5.4×10^5^ ± 0.8×10^5^, *n* = 59/7 fields/individuals) compared to controls (controls: 1.7 × 10^5^ ± 0.3 × 10^5^ *n* = 65/6 fields/individuals; *p* < 0.01; Figure 2B). Moreover, as in the brain TDP-43 positive protein aggregates in ALS and frontotemporal dementia often contain p62 protein (Tanji et al., 2012), we evaluated the consistent presence of p62 in TDP-43 pathological inclusions in ALS patients retina (Figure 2C). We found a positive co-localization between p62 and TDP-43 immunostaining in both ALS patients and age matched control. Strikingly, the Manders coefficient of positive colocalization was higher in ALS patients (ALS: 0.80 ± 0.03, *n* = 17/4 fields/individuals) respect to age matched controls (controls: 0.70 ± 0.03, *n* = 17/4 fields/individuals; *p* < 0.05; Figure 2D).

These findings show an increase in p62 and TDP-43 inclusions in the retina of ALS patients with respect to controls thus pointing at them as putative biomarkers for ALS diagnosis.

### 3.2. Retinal ganglion cell layer of ALS patients exhibits increased microglia density and activation of apoptosis

**Figure 3:**
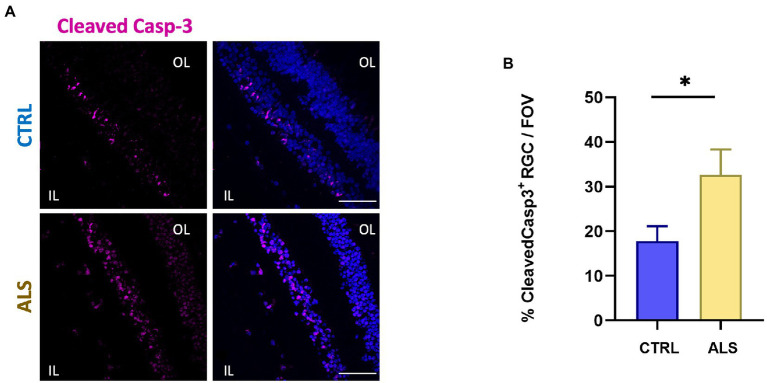
Human ALS retina show neurodegeneration in the Ganglion Cell Layer. **(A)** Representative immunostaining of anti-cleaved Caspase-3 (magenta) and Hoechst for nuclei visualization (blue) in retinal slices from ALS patients and control individuals; scale bar 50 μm. **(B)** Bar chart displaying the percentage of cleaved Caspase-3 positive cells in the ganglion cell layers (IL, inner layer; ^*^p < 0.05 ALS vs. CTRL; Mann–Whitney U test; ALS *n* = 50/5 fields/individuals; CTRL *n* = 51/6 fields/individuals).

Increased cleavage of proteins operated by Caspase-3, has been associated with apoptosis activation and neurodegeneration in ALS (Pasinelli et al., 2000; Rojas et al., 2021). We previously reported the immunoreactivity for the cleaved Caspase-3 at the level of the retinal ganglion cell (RGC) layer of the AD human retina (Grimaldi et al., 2019). We here report that the positive staining for cleaved Caspase-3 is also present in ALS human retinas (Figure 3A, magenta dots) in the RGC layer. In particular, we found that the percentage of RGCs positive for cleaved Caspase-3 in each field of view (FOV) examined was increased compared to age matched controls (ALS: 33 ± 6%, *n* = 50/5 fields/individuals; CTRL: 18 ± 3%, *n* = 51/6 fields/individuals; *p* < 0.05; Figure 3B). These results indicated that in the ALS retinal the inner layer (IL) is more subject to apoptosis activation, thus suggesting that visual defects observed in ALS may rely on RGC neurodegeneration.

**Figure 4:**
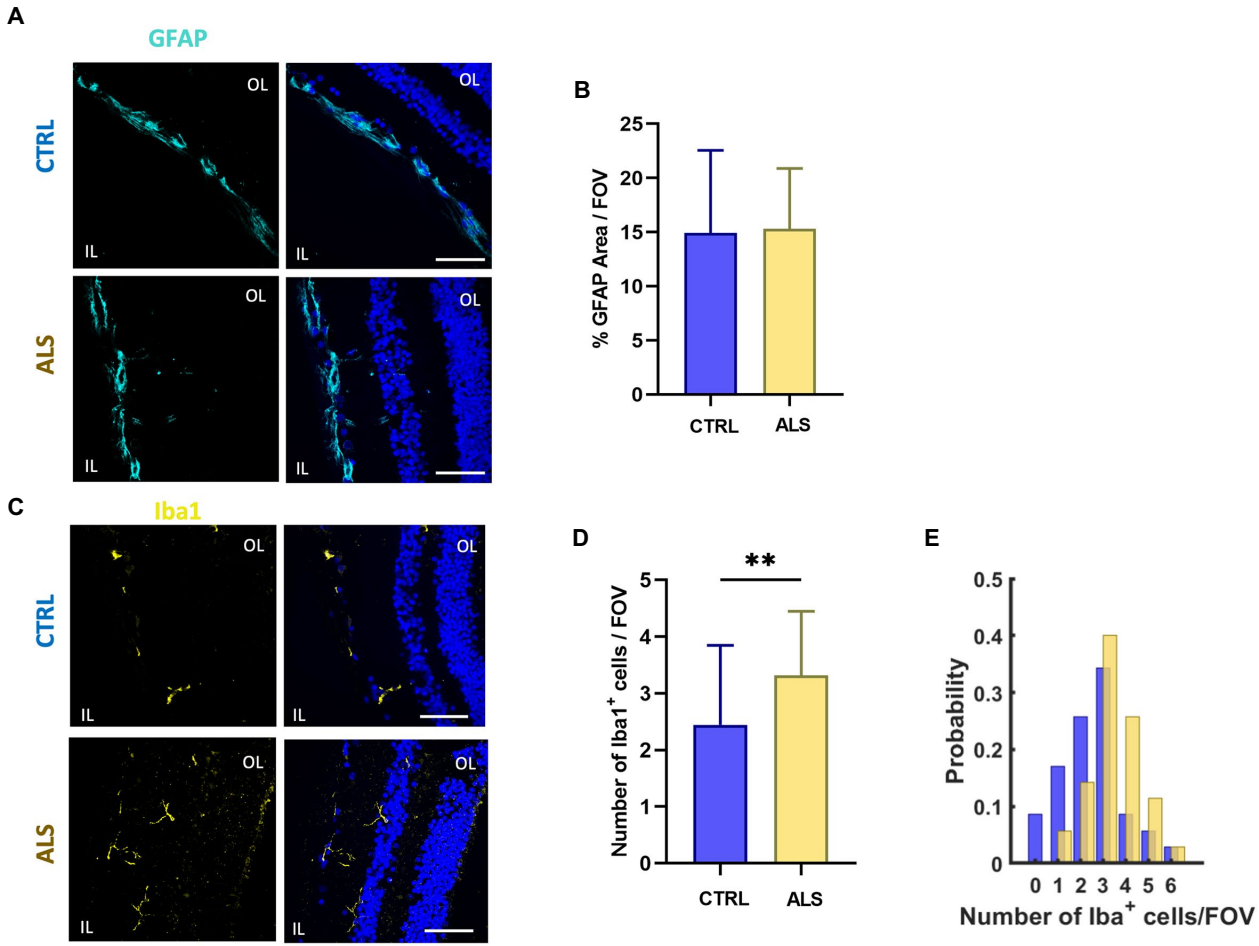
Astrocyte and microglia reactivity is not altered in ALS human retina. **(A)** Representative images of retinal slices from ALS patients and healthy controls immunolabeled with anti-GFAP (cyan); Hoechst was used for nuclei visualization (blue); scale bar 50 μm. **(B)** Bar chart showing the percentage of the area covered by GFAP signal/field of view (ALS: *n* = 20/4 fields/patients; CTRL *n* = 18/4 fields/patients). **(C)** Representative images of retinal slices from ALS patients and control cases immunolabeled with anti-Iba1 antibody (yellow) and Hoechst for nuclei visualization (blue); scale bar 50 μm. **(D)** Bar graph displaying the number of Iba1+ cells for each field of view (ALS *n* = 35/6 fields/patients; CTRL *n* = 36/6 fields/patients; ^**^p < 0.01). **(E)** Distribution of the number of retinal microglia cells in each field of view (400 μm^2^) in ALS patients (yellow) and age matched controls (blue).

In ALS spinal cord, reactive astrocytes and microglia have been found closely associated with dying motoneurons suggesting a role for glial cells in the ALS neuropathological process (Appel et al., 2011; Van Harten et al., 2021). To evaluate putative astrocytes and microglia reactivity in the retina of ALS patients, we performed immunostaining for GFAP and Iba1, respectively. In contrast to that reported in other neurodegenerative diseases (such as AD), the analysis of confocal images did not reveal signs of astrocytes reactivity in ALS retina, evaluated as the area covered by the GFAP signal (Figures 4A,B). Conversely, immunostaining against Iba1, revealed that microglia cell density was increased in ALS patients’ retina compared to age matched controls (ALS: 3.3 ± 0.2; *n* = 35/6 fields/patients; CTRL: 2.4 ± 0.2, *n* = 36/6 fields/controls; *p* < 0.01; Figures 4C–E).

Altogether these results confirming the presence of protein inclusions, increased microglia density, and retinal cell degeneration in human ALS retina suggest the possibility of defining a set of retinal biomarkers to assist with ALS diagnosis and monitoring.

## 4. Discussion

We report for the first time, the presence of cytoplasmic inclusions of TDP-43 and p62 in retinal cells along with degeneration of neurons in the retina of sporadic ALS patients.

Recently, many studies have reported decreased retinal nerve fiber layer (RNFL) thickness associated with various neurological and neurodegenerative diseases. This has been observed in AD, minimal cognitive impairment (Ono et al., 1988; Howland, 1990; Philippou and Joubert, 2013; Bozzoni et al., 2016), multiple sclerosis (Pugdahl et al., 2007; Sharma et al., 2011), Parkinson’s disease (Moss et al., 2012), diffuse Lewy body disease (DLB; Cerveró et al., 2019), and Wilson’s disease (Yap et al., 2019). Furthermore, in AD, the retina shows the presence of amyloid beta protein and tau aggregates, neurodegeneration, glial reactivity, and altered vascularity (Gupta et al., 2021). Therefore, the possibility of using the eye as a window to the CNS has prompted further research into the presence of protein aggregates, typical of other neurodegenerative disorders, in the retina of patients and animal disease models. In particular, for ALS cases, typical motor neuron inclusions are positive for ubiquitin/p62, ubiquilin-2, SOD1 (Soldatov et al., 2021), TDP-43, and pTDP-43 (Arai et al., 2006; Neumann et al., 2006).

However, retinal involvement of ALS-related protein aggregates has only been reported in one fALS patient with the C9orf72 mutation and in two fALS mouse models to date. Specifically, SOD1 inclusions have been found in *hSOD1G93A* mice (Soldatov et al., 2021), in which retinal cell degeneration and protein aggregates have been reported to parallel spinal cord degeneration (Hashizume et al., 2008). In addition, retinal inclusions of p62 were observed in the inner layer of *C9orf72* mouse retina. In contrast, the ALS *FUS* mouse model showed no significant structural retinal changes (Soldatov et al., 2021). We observed, for the first time, in the retinas of sALS patients, the presence of TDP-43 and p62 co-localized inclusions at the level of ganglion cells and inner nuclear layers. Notably, while the nuclear expression of TDP-43 was similar in ALS patients compared with age-matched controls, its cytoplasmic localization was present only in ALS patients.

This is consistent with the idea that the inner layer may be the site of ALS-associated neurotoxicity (Rojas et al., 2020). Given that histopathological analysis of the retina from a C9-ALS patient showed p62-positive intracytoplasmic perinuclear inclusions in the IL similar to those observed in the dentate gyrus of ALS patients with the C9orf72 mutation. Notably, in line with what reported in motoneurons (Arai et al., 2003; Nakano et al., 2004; Mizuno et al., 2006; Tanji et al., 2012; Foster et al., 2021), we observed a strong co-localization of p62 and TDP-43 staining in RGC. Indeed, cytoplasmic aggregates of TDP-43-positive proteins also contain the p62 protein. We can hypothesize that, similar to what occurs in motoneurons, these inclusions may comprise different protein components, such as neurofilaments and RNA (Blokhuis et al., 2013).

We also reported the presence of retinal ganglion cell degeneration in sALS patients, evidenced by cleaved Caspase-3 positivity of RGC neurons (TuJ1-positive cells). This is in line with the RNFL thinning in ALS patients recently demonstrated by optical coherence tomography (Rohani et al., 2018).

We also, evaluated the glial reactivity in retinal slices as, among the multifactorial mechanisms underlying ALS-associated neurodegeneration, inflammation is one of the most prominent hallmarks of sporadic and familial forms of ALS (Liu and Wang, 2017), with overall reactivity of astrocytes and microglia and overproduction of inflammatory cytokines (Peric et al., 2017; Clarke and Patani, 2020; Vahsen et al., 2021; Van Harten et al., 2021).

It should be noted that we found no signs of astrocyte reactivity in the retina of ALS patients contrary to expectations. On the other hand, counting microglia cells, we found increased microglia density in ALS retina.

It is possible, however, that the glial phenotype is underestimated in this context. Indeed, the use of coronal retinal slices prevented a morphological analysis of astrocytes and microglia because of the extremely thin thickness of the slices. In addition, inclusion in paraffin prevented a Real Time PCR analysis.

In conclusion, our data support the idea that although ALS has traditionally been considered a disorder purely of the motor system, several non-motor systems, including the eyes, are involved and affected by the disease. Furthermore, the presence of protein aggregate inclusions in the retina could become a new fingerprint panel to characterize ALS, supporting the possibility of using ocular biomarkers for early diagnosis of ALS-associated neurodegeneration.

Indeed, to date, there is a lack of reliable and noninvasive clinical assessment to monitor ALS patients and document response to therapy in ALS clinical trials. Measurement of retinal changes could become an inexpensive and noninvasive method to assess neurodegeneration and, given its correlation with disease severity, can be used to assess disease progression in ALS patients (Soldatov et al., 2021).

However, this will require the development of specific ligands for ALS biomarkers and remote high-resolution imaging techniques to achieve noninvasive and inexpensive diagnosis and monitoring of ALS by scanning the retina.

## Data availability statement

The raw data supporting the conclusions of this article will be made available by the authors, without undue reservation.

## Ethics statement

The use of Human tissue has been approved by the Ethics Committee of Fondazione Santa Lucia I.R.C.C.S. to GR. Written informed consent was not provided because we used tissues obtained from a tissue bank.

## Author contributions

NP, YG, SC, and MP designed, carried out, and analyzed the immunofluorescence experiments on protein aggregates and neurodegeneration. LM designed, carried out, and analyzed the immunofluorescence experiments on astrocytes and microglia. NP and YG designed, carried out, and analyzed the confocal acquisitions. SG designed and wrote the Matlab-based code. SDA wrote the manuscript with the help of GR and SG and conceived, supervised, and administered the project. SDA and GR acquired funds for the project. All authors contributed to the article and approved the submitted version.

## Funding

This research was funded by the REGIONE LAZIO (19036AP000000019 and A0112E0073; to SDA); Fulbright (FSP-P005556; to SDA); Rome Technopole PNRR, FP7 (to SDA); ERC-2019-Synergy Grant (ASTRA, n. 855923; to GR); EIC-2022-PathfinderOpen (ivBM-4PAP, n. 101098989; to GR). This research was partially funded by grants from Sapienza University (to SDA).

## Conflict of interest

YG and MP are employed by D-Tails s.r.l. SDA is a scientific advisor of D-Tails s.r.l.

The remaining authors declare that the research was conducted in the absence of any commercial or financial relationships that could be construed as a potential conflict of interest.

## Publisher’s note

All claims expressed in this article are solely those of the authors and do not necessarily represent those of their affiliated organizations, or those of the publisher, the editors and the reviewers. Any product that may be evaluated in this article, or claim that may be made by its manufacturer, is not guaranteed or endorsed by the publisher.
